# Etiologies des pleurésies exsudatives: à propos de 424 cas à Madagascar

**DOI:** 10.4314/pamj.v9i1.71213

**Published:** 2011-07-31

**Authors:** Joëlson Lovaniaina Rakotoson, Radonirina Lazasoa Andrianasolo, Robert Jocelyn Rakotomizao, Marie Danielle Hanta Vololontiana, Kiady Ravahatra, Jobeline Rajaoarifetra, Christophe Félix Ange Andrianarisoa

**Affiliations:** 1 Unité de soins, de formations et de recherches de Pneumologie du Centre Hospitalier Universitaire d'Antananarivo, Madagascar; 2 Unité de soins, de formations et de recherches de Maladies Infectieuses du Centre Hospitalier Universitaire d'Antananarivo, Madagascar; 3 Unité de soins, de formations et de recherches de Médecine Interne du Centre Hospitalier Universitaire d'Antananarivo, Madagascar

**Keywords:** Tuberculose pleurale, biopsie pleurale, tumeurs pleurales, pleurésies d'exsudatives

## Abstract

**Introduction:**

La pleurésie constitue un motif fréquent de consultation en pneumologie. Notre travail a pour objectif de déterminer les étiologies des pleurésies exsudatives afin d'en faciliter les démarches étiologiques.

**Méthodes:**

Il s'agit d'une étude rétrospective réalisée chez des patients ayant une pleurésie exsudative et bénéficiant une biopsie pleurale à l'aveugle à l'aide de l'aiguille de Castelain, pendant une période de 5 ans (2005 à 2009).

**Résultats:**

Parmi les 424 patients inclus, 259 hommes (61,08%) et 165 femmes (38,91%) étaient individualisés. Les pleurésies étaient d'origine tuberculeuse dans 298 cas (70, 28%), métastatique dans 63 cas (14,85%), inflammation non spécifique dans 51 cas (12,02%). Des fibres musculaires striées étaient biopsiées dans 12 cas (2,83%).

**Conclusion:**

La biopsie pleurale occupe une place prépondérante dans la recherche étiologique des pleurésies d'exsudatives à Madagascar où la tuberculose sévit encore en mode endémique.

## Introduction

Bien que la thoracoscopie constitue l'examen de choix pour explorer la plèvre, la biopsie pleurale occupe encore une place prépondérante dans les pays en développement. A Madagascar, seule la biopsie pleurale à l'aveugle constitue un moyen d'exploration des pleurésies exsudatives car notre centre hospitalier universitaire (CHU) n'a pas encore à sa disposition une thoracoscopie. C'est pour cela que nous avons essayé d’établir les profils étiologiques des pleurésies exsudatives à partir d'une biopsie pleurale à l'aveugle afin d'améliorer leur prise en charge.

## Méthodes

Nous avons menés une étude rétrospective allant de janvier 2005 à décembre 2009 sur les résultats des examens anatomo-pathologiques d'une biopsie pleurale chez les malades porteurs de pleurésie exsudative. Le caractère exsudatif des pleurésies a été affirmé par la présence du taux de protéine dans le liquide pleural supérieur ou égal à 30 grammes par litre. Tous les malades ayant des pleurésies exsudatives ont bénéficié une biopsie pleurale à l'aveugle à l'aide d'une aiguille de Castelain et les prélèvements obtenus ont été analysé.

## Résultats

Quatre cent vingt-quatre (424) cas de pleurésie exsudative ont été inclus dans notre étude. Nos effectifs étaient composés de 259 hommes (61,08%) et 165 femmes (38,91%) avec un sex ratio de 1,5. La moyenne d’âge était de 46,59 ans. La sérologie VIH était négative dans tous les cas. Les étiologies (Tableau 1) étaient constituées par 298 cas de tuberculose (70,28%), de 63 cas de métastase pleurale (14,85%) dont 30 cas de métastase d'adénocarcinome, 15 cas de carcinome épidérmoïde, 4 cas de lymphome malin non Hodgkinien, 2 cas de sarcome, 2 cas de carcinome papillaire, métastase de cancer de la thyroïde (2 cas), prostate (1 cas), rein (1 cas), digestif (1 cas), bronchique (2 cas), sein (1 cas), ovaire (1 cas). Cinquante et un cas d'inflammation non spécifique (12,02%) et 12 cas de fibres musculaires striés (2,83%) ont été constatés au cours de cette étude.

**Tableau 1:** étiologies des pleurésies exsudatives à Madagascar

Etiologies	Nombre	Pourcentage
Tuberculose	298	70,28
		
**Métastases**	**63**	**14,85**
Adénocarcinome	30	47,61
Carcinome épidérmoïde	15	23,80
LMNH	4	6,34
Fibrosarcome	2	3,17
Carcinome papillaire	2	3,17
Cancer thyroïde	2	3,17
Cancer digestif	2	3,17
Cancer bronchique	2	3,17
Cancer prostate	1	1,58
Cancer rein	1	1,58
Cancer sein	1	1,58
Cancer ovaire	1	1,58
		
**Inflammation non spécifique**	**51**	**12,02**
**Fibre musculaire strié**	**12**	**2,83**

LMNH : Lymphome Malin Non Hodgkinien

## Discussion

La tuberculose occupe la première place parmi les étiologies des pleurésies exsudatives à Madagascar, suivie des métastases de cancers avec 9 lésions primitives identifiées. Les résultats de notre étude semblent identiques à ceux des pays à forte prévalence tuberculeuse. La biopsie pleurale à l'aveugle, malgré son faible rendement en cas de pleurésies cancéreuses, tient une place particulière dans l'exploration étiologique des pleurésies exsudatives dans les pays à faible revenu comme Madagascar car notre CHU n'est pas encore équipé de pleuroscopie. Au Maroc, Berrada et al retrouvaient 251 cas de pleurésies tuberculeuses sur une période de 6 ans dont le diagnostic était confirmé par l'histologie dans 58% des cas [1]. Dans son étude sur 200 cas de pleurésie, Asriri et al réalisaient la ponction biopsie pleurale dans 84% des cas et ont constaté une étiologie tuberculeuse dans 52,5% des cas, bactérienne dans 7% des cas, néoplasique dans 4,5% des cas [2]. Bakhatar et al retrouvaient sur 104 cas de pleurésie 36% de cas de tuberculose, 12% de métastases, un cas de localisation pleurale d′une leucémie lymphoïde chronique [3] et 8% d’étiologie bactérienne [3]. Les métastases constituent la grande majorité des tumeurs pleurales [4]. Elles représentent la première cause d’épanchement pleural après 50 ans [4]. Le point de départ est le plus souvent thoracique, et 60% des métastases pleurales ont pour origine un cancer broncho-pulmonaire ou un cancer du sein [4]. Elles sont surtout représentées par les métastases des adénocarcinomes, des carcinomes malpighiens, des carcinomes à petites cellules, ou encore des mélanomes [5]. Les tumeurs primitives de la plèvre les plus fréquentes sont les tumeurs solitaires fibreuses, les mésothéliomes et les sarcomes, les tumeurs malignes constituant seulement 2 à 3% de toutes les tumeurs pleurales [5]. Aucune lésion tumorale primitive de la plèvre n'a été identifiée au cours de notre étude. Idéalement, les prélèvements histologiques sont obtenus par biopsie pleurale à l'aveugle, sous pleuroscopie, ou guidée par tomodensitométrie. La biopsie pleurale guidée par tomodensitométrie, à l'aiguille biopsique automatique, a une valeur prédictive positive de 100%, une valeur prédictive négative de 75% et une précision de 91% pour le diagnostic de malignité dans la série d'Adams [4]. Mais malgré notre expérience en matière de biopsie pleurale à l'aveugle, qui est également opérateur dépendant, 12 cas de fibres musculaires striés (2,83%) ont été prélevés.

## Conclusion

La biopsie pleurale à l'aveugle peut être proposée devant le bilan étiologique d'un épanchement pleural exsudatif compatible avec une tuberculose (pays à incidence élevée, épanchement lymphocytaire, intra-dermo-réaction à la tuberculine positive) ou avec une pathologie néoplasique déjà identifiée.
